# Nutritional Quality, Fatty Acids Profile, and Phytochemical Composition of Unconventional Vegetable Oils

**DOI:** 10.3390/molecules30153269

**Published:** 2025-08-04

**Authors:** Wiktoria Kamińska, Anna Grygier, Katarzyna Rzyska-Szczupak, Anna Przybylska-Balcerek, Kinga Stuper-Szablewska, Grażyna Neunert

**Affiliations:** 1 Department of Physics and Biophysics, Faculty of Food Science and Nutrition, Poznan University of Life Sciences, Wojska Polskiego 38/42, 60-637 Poznan, Poland; wiktoria.kaminska@up.poznan.pl; 2 Department of Food Technology of Plant Origin, Faculty of Food Science and Nutrition, Poznan University of Life Sciences, Wojska Polskiego 31, 60-634 Poznan, Poland; anna.grygier@up.poznan.pl; 3 Department of Chemistry, Faculty of Forestry and Wood Technology, Poznan University of Life Sciences, Wojska Polskiego 75, 60-625 Poznan, Poland; katarzyna.rzyska@up.poznan.pl (K.R.-S.); anna.przybylska@up.poznan.pl (A.P.-B.); kinga.stuper@up.poznan.pl (K.S.-S.)

**Keywords:** fatty acids profile, nutritional quality, bioactive compounds, oxidation stability

## Abstract

This study compares the nutritional and metabolic properties of unconventional cold-pressed vegetable oils available on the Polish market. Twelve oils—milk thistle, evening primrose, flaxseed, camelina sativa, black cumin, pumpkin seed, sesame, mustard seed, sea buckthorn, blue poppy seed, borage, and safflower—were examined. The chosen oils were investigated based on their fatty acids profiles, total phenolic compounds (TPC), tocopherols, and pigment contents. Despite the high polyunsaturated fatty acids (PUFAs) content raising concerns about oxidative stability, the significant tocopherol levels and polyphenols content contribute to antioxidative protection. These oils’ favorable hypocholesterolemic, antiatherogenic, and antithrombogenic properties were highlighted by key nutritional indices, showing potential benefits for cardiovascular health. These results suggest that these oils are a promising dietary supplement for promoting both cardiovascular health and sustainability, owing to their rich content of essential fatty acids and bioactive compounds. Moreover, high correlations were found between theoretical and experimental established oxidative stability of the tested oils at the ending stage of the thermostat test.

## 1. Introduction

Fatty acids are essential components of the human diet worldwide. They are classified as saturated (SFAs), monounsaturated (MUFAs), or polyunsaturated (PUFAs) based on their degree of saturation. In recent years, dietary changes aligned with healthy eating principles have led to a reduction in the intake of SFAs and an increase in the consumption of MUFAs and PUFAs [1]. Fatty acids are obtained from various dietary sources, each with a characteristic fatty acids profile (FAME), which consequently influences health outcomes. From this perspective, the composition of fatty acids should be assessed to determine their nutritional and/or medicinal value, especially in fatty-acid-rich foods such as edible oils, wherein the fatty acids constitute over 95% of their composition [2]. This trend has driven a growing demand for alternative vegetable oils that are low in SFAs and high in MUFAs and PUFAs.

To determine the health and nutritional properties of foods rich in fatty acids, many indexes have been defined, which can be divided into several basic subcategories depending on the purpose for which they were developed, such as qualitative, nutritional, lipid-related, or energy-related indexes [3,4]. The most commonly used indexes include the PUFA/SFA and omega-6/omega-3 (n-6/n-3) or n-3/n-6 ratios. More specific indexes consider the role of individual fatty acids in greater detail, particularly in the prevention of cardiovascular diseases or susceptibility to oxidation. These include the atherogenicity index (AI), thrombogenicity index (TI), hypocholesterolemic/hypercholesterolemic ratio (h/H), unsaturation index (UI), and oxidizable value (Cox) [5,6]. Not only the dietary content of fatty acids but also their endogenous metabolism, e.g., via elongation and desaturation, affects their impact on the body [7,8]. For metabolic indicators, in order to determine possible metabolic pathways of fatty acids and quantify the activity of desaturases and elongases, a variety of indices are used [3,4]. The most frequently calculated include elongase and desaturase indexes as well as linoleic desaturation (LDR) and oleic desaturation (ODR) ratios [9]. All of these indexes are determined based on the composition of different fatty acid groups and the number of double bonds, for the determination of which FAME is the base.

The PUFA/SFA ratio is one of the most frequently used indicators for assessing the impact of diet on cardiovascular health (CVH). It is generally assumed that PUFAs in the diet can lower low-density lipoprotein (LDL) cholesterol levels and reduce total serum cholesterol, while SFAs contribute to higher serum cholesterol levels [10,11]. As a result, a higher PUFA/SFA ratio indicates a more favorable impact on health, whereas a low PUFA/SFA ratio in the diet (below 0.45) is considered a risk factor for elevated blood cholesterol levels [12]. In addition, some MUFAs, such as oleic acid (C18:1 n-9*cis*), can also impact CVH by increasing the activity of low-density lipoprotein receptors (LDLRs) and lowering serum cholesterol concentrations. On the other hand, some studies report that not all major classes of PUFA have a positive effect on the prevention of cardiovascular disease, for example, linoleic acid (LA, C18:2 n-6), the intake of which in the diet is inversely correlated with this activity [13].

The composition of fatty acids not only is important for determining the nutritional value of oils, but it also has a critical impact on their oxidative stability [14,15]. Sensitivity to oxidation increases exponentially with the number of double bonds per fatty acid molecule. Therefore, the oxidation rates of α-linolenic acid (ALA, C18:3 n-3) and LA are about 100 and 70 times faster, respectively, than that of C18:1. The oxidation rates for EPA and DHA are even higher—approximately 250 to 350 times greater [16]. Hence, the PUFA/SFA ratio (also known as the polyene index) can be used as a measure of the degree of polyunsaturation in oils and oil blends and their tendency to undergo auto-oxidation [5]. So, a higher proportion of SFAs is favorable to more oxidative stability, while a higher proportion of PUFAs is more desirable from the consumer’s point of view.

In addition to fatty acids, cold-pressed vegetable oils are a rich source of lipid-soluble phytochemicals such as tocopherols, phytosterols, and polyphenols. These compounds have been shown to exhibit strong antioxidant properties and are increasingly recognized for their role in protecting oils from oxidation and providing additional health benefits [17]. Tocopherols, especially the α- and γ-homologs, act as natural radical scavengers, while polyphenols and sterols contribute to anti-inflammatory, hypocholesterolemic, and potentially anticancer effects. Due to their functional significance, these phytochemicals are now considered essential markers of oil quality.

It is possible to evaluate oil stability under accelerated storage conditions, during which periodic analyses are performed to monitor chemical, physical, or sensory changes. The Schaal oven test, a thermostatic test, is one of the most widely used methods [18]. This test makes it possible to know the oil shelf life, since the results provided have a good correlation with the evaluation carried out in storage at room temperature [19].

The aim of the present research study was to characterize and compare the nutritional indices and oxidative stability of twelve edible cold-pressed oils—linseed (FSO), milk thistle (MTSO), camelina seed (CSSO), pumpkin seed (PSO), black cumin (BCSO), evening primrose (EPSO), sesame (SO), mustard seed (MSO), sea buckthorn (SBO), blue poppy seed (BPSO), borage (BSO), and safflower (SFO) oils—based on their fatty acid composition. Moreover, their metabolic indices were evaluated. Additionally, the composition of tocopherols, polyphenols, and pigments, which influence the quality and health benefits of the aforementioned oils, was characterized. In a further step, the research focused on determining the correlation between the calculated theoretical oxidizable indices and the oxidation rate determined based on the Schaal oven test.

## 2. Results and Discussion

### 2.1. Fatty Acid Composition and Nutrient Value Indices

#### 2.1.1. The Fatty Acids Profile (FAME) and Qualitative Indexes

Fatty acid composition is critical in defining the qualitative and nutritional characteristics of vegetable oils. FAMEs for SO, MSO, SBO, BPSO, BSO, and SFO are shown in Table 1. A detailed analysis of the fatty acid content for the remaining oils (MTSO, EPSO, FSO, CSSO, BCSO, PSO) was performed in our earlier study [20]. Based on the FAMEs, the sums of SFAs and unsaturated fats (UFAs), as well as MUFAs and PUFAs and the PUFA/SFA and n-6/n-3 ratios, were determined and are listed for all tested oils in Table 2.

The studied oils had characteristic FAMEs that were generally similar to those described in the literature [21,22,23,24,25]. Variations in the fatty acid composition of individual oils can occur even within the same plant species due to genetic diversity, geographical and climatic conditions, harvest time, seasonality, and production techniques [26,27].

Significant differences in FAME were observed among the studied oils. It was found that the SFA content ranged from 6.07% to 21.18%, with the highest value found in PSO. The predominant SFA in all oils, except MTSO, was palmitic acid (C16:0), ranging from 3.48% in MSO to 12.60% in PSO. In MTSO, the main SFA was stearic acid (C18:0) (4.43%), which was also present in a few percent concentration in the other oils. SFAs have been associated with high levels of cholesterol in blood [28]. However, not all SFAs have the same effect on serum cholesterol. Lauric acid (C12:0), myristic acid (C14:0), and C16:0 can increase serum cholesterol concentrations by inhibiting the activity of LDL receptors, whereas C18:0 was found to be biologically neutral and had no effect on circulating LDL cholesterol levels [3].

Substituting SFAs with UFAs or proteins helps lower cholesterol levels, which contribute to atherosclerosis [13]. The analyzed oils exhibited a high UFA content (> 77%), with particularly high levels noted in MSO, SBO, and SFO (93.92%, 90.17%, and 89.33%, respectively). Among UFAs, PUFAs were predominant in most cases. An exception was MSO, where MUFAs dominated, primarily due to the high level of C18:1 (48.88%). C18:1, which was also the foremost acid among MUFAs in all tested oils, can positively impact CVH by increasing the activity of LDL receptors [3].

In accordance with guidelines established by the Food and Agriculture Organization (FAO) and the World Health Organization (WHO), PUFAs are recognized as critical components in the prevention of cardiovascular diseases [13]. Therefore, the PUFA/SFA ratio is the most commonly used index for evaluating the medical/health value of dietary foods as an index to assess the impact of diet on CVH. Food products with a low PUFA/SFA ratio (less than 0.45) are generally not recommended for human consumption due to their potential to increase blood cholesterol levels. Simultaneously, the higher the ratio, the more positive its predicted effect on health. Among the studied oils, the highest parameter value was found in MSO (7.02) (Table 2), which can be attributed to the lowest SFA content, although its PUFA amount was also the lowest among the tested oils. Slightly lower PUFA/SFA values were obtained for SFO (6.86) and BPSO (6.60), which contained the most PUFAs. The lowest PUFA/SFA ratios were observed in PSO (2.51), SO (2.94), and FSO (3.42), which had the highest SFA contents.

Among PUFAs, LA and ALA are classified as essential fatty acids (EFAs) due to their inability to be synthesized de novo in the human body and their critical role in modulating lipid profiles, reducing systemic inflammation, and decreasing the incidence of cardiovascular disease [13]. In the studied oils, ALA was the primary PUFA detected in FSO and CSSO, at levels of 46.73% and 52.70%, respectively. Similar results for FSO were reported in [21], where ALA accounted for 43.67% of total fatty acids, while the amount of this acid determined in CSSO exceeded previously reported values [29,30]. The high proportion of ALA in FSO and CSSO resulted in the lowest n-6/n-3 ratios, 0.31 and 0.13, respectively, corroborating previous reports [12,31], whereas LA was the main PUFA in the remaining oils, ranging from 37.52% in BSO to 72.74% in SFO, which was mostly several dozen times higher than the ALA content in these oils. The low level of ALA detected in most samples resulted in high n-6/n-3 ratios, reaching up to 292.9 for EPSO (Table 2), although still lower than that found for poppy seed oil (364) by Strati et al. [32]. In our study, however, BPSO was characterized by an n-6/n-3 ratio over seven times lower (52.67) than that cited in the above report. A relatively high n-6/n-3 index was also obtained for SO and SFO, 146.03 and 142.62, respectively, much higher the value found for sesame oil (9.5) by Hashempour-Baltork et al. [12]. These extremely high n-6/n-3 ratios, especially in EPSO and SFO, may pose a long-term pro-inflammatory risk if consumed as the sole source of dietary fat. To mitigate this, it is advisable to blend such oils with n-3-rich oils like FSO or CSSO in order to achieve a more favorable fatty acids profile, in line with WHO dietary recommendations (5:1 to 1:1) [33].

A high ratio of n-6 to n-3 fatty acids in the diet increases the risk of many diseases, including cancer. Therefore, it is important to maintain an appropriate balance between n-6 and n-3 acids, which, according to WHO recommendations, should be between 5:1 and 1:1 for the general population and below 2:1 for high-risk groups [31,32]. Among the analyzed oils, only MTSO and MSO were shown to have a beneficial n-6/n-3 ratio (3.36 and 2.8, respectively), although previously published data reported higher values of this parameter for MTSO [23,31,32]. MSO also showed the highest PUFA/SFA ratio, which confirms its health-promoting potential. Nowadays, typical Western diets are characterized by excessive n-6 consumption. Hence, introducing oils with a significant content of n-3 acids (such as FSO and CSSO) into the diet could help correct the deficiency of n-3 acids and have a beneficial effect on human health.

It should also be noted that small percentages of erucic acid (C22:1) were detected in MSO (2.35%), BSO (3.08%), and CSSO (4.96%). Some reports have also indicated its presence in other unconventional oils, such as sesame oil [25]. C22:1 is an undesirable fatty acid in edible oils, as it has a negative impact on health. This monounsaturated fatty acid is associated with fatty degeneration of the heart and liver and probably with inhibiting the body’s growth [34]. The maximum allowable content of C22:1 in total fatty acids is 5% for camelina, mustard, and borage oils and 2% for other plant oils in the European Union (Council Directive 2019/1870), as well as 2% for all oils in the United States, Australia, and New Zealand [35]. As indicated in the literature, the content of C22:1 in plant oils may vary widely, especially in mustard oil or traditional rapeseed oil, where it can reach up to 50% [36]. In the studied oils, the amount of C22:1 was within the allowable range (except in CSSO), which may be related to the origin or genotype of the plants used for the oils’ production [35]. Therefore, the oils mentioned above can be considered safe for human consumption, although the potential presence and content of erucic acid in oils should be taken into account when designing a healthy diet.

#### 2.1.2. Nutritional and Metabolic Indexes

Indices such the sum of SFAs, MUFAs, PUFAs, the PUFA/SFA ratio, or the n-6/n-3 ratio are classical indicators of the nutritional and/or medicinal value of dietary fats. However, these indices are too general and are unsuitable for assessing the atherogenic or thrombogenic potential of foods. In recent years, indexes more useful than simple fatty acid composition for evaluating the nutritional quality of oils, such as atherogenicity (AI), thrombogenicity (TI), and the hypocholesterolemic/hypercholesterolemic ratio (h/H) have been used frequently to characterize edible oils and their blends [3,5,6,37]. The calculations of the above-mentioned nutritional indices of the studied oils are listed in Table 2.

The AI reflects the relationship between the sum of SFAs, excluding C18:0, and the sum of UFAs, indicating the balance between pro- and antiatherogenic fatty acids. The TI, on the other hand, characterizes the thrombogenic potential of fatty acids, i.e., the relationship between pro- and antithrombogenic fatty acids [28]. Both AI and TI serve as valuable indicators for assessing the potential impact of fatty acid composition on CVH. From a nutritional standpoint, it is recommended that these indices remain at low levels, with AI values below 1.0 and TI values below 0.5, in order to minimize cardiovascular risk [12]. The h/H index describes the relationship between hypo- and hypercholesterolemic fatty acids. Therefore, the h/H ratio may more accurately reflect the impact of fatty acid composition on cholesterol levels compared with the PUFA/SFA ratio, and high h/H values are desirable for nutritional purposes [3,38].

The AI values of all oils ranged from 0.04 (for MSO) to 0.18 (for PSO), which are well below the given criterion. A similar low AI value was provided by Qian et al. [6] for several unconventional cold-pressed oils, including poppy seed (0.18), black cumin (0.15), safflower (0.11), borage (0.18), and milk thistle seed oils (0.23). Comparable values were also obtained for pumpkin seed oil (0.18) by Siol et al. [5] and for sesame oil (0.13) by Hashempour-Baltork et al. [12], although the AI value can be significantly higher for some oils, as was shown by Ulbricht and Southgate for coconut oil (13.63) [28]. The second nutritional index, TI, was below the recommended level for almost all oils (ranging from 0.05 to 0.35), corroborating previous reports for cold-pressed oils rich in PUFAs [6,12,30,37]. The exception was PSO, which exceeded the recommended value, with a TI of 0.52. It was also characterized by the lowest h/H ratio (6.0), comparable to the TI and h/H values obtained for pumpkin seed oil by Siol et al. [5]. In turn, the highest h/H ratio was calculated for MSO (26.28), which was higher than that found in apricot oil (21.49) by Qian et al. [6] but lower than the value reported for raspberry oil (41–49) [39]. The h/H ratios of the remaining tested oils ranged from 7.06 (for FSO) to 14.65 (for SBO), suggesting a desirable balance between hypocholesterolemic PUFAs and hypercholesterolemic fatty acids, offering positive health effects. Plant oils characterized by low AI and TI values and a relatively high h/H ratio, when included in the diet, may contribute to lowering cholesterol and be associated with better CVH [40].

Vessby et al. reported that calculated activities of enzymes such as 9-desaturase, among others, can be used as substitutes for laboratory measurements of desaturase activity [41]. Therefore, FAME was also used to estimate certain metabolic ratios in order to assess fatty acid biosynthesis. Metabolic indicators such as elongase and desaturase (Δ9-desaturase (C18:1)) indexes, as well as linoleic (LDR) and oleic (ODR) desaturation ratios, were determined using an enzymatic approach called the “products/substrate ratio” [4], and are presented in Table 2. Elongation converts C16:0 into C18:0, whereas desaturation adds double bonds to SFAs, converting them into MUFAs.

The elongase index exhibited considerable variation among the tested oils, ranging from 2.95 to 65.87, with the highest value noted for PSO. Oils with low elongase values, especially CSSO (4.26) and BCSO (2.95), were poor in C18:0 and possessed similarly high Δ9-desaturase (C18:1) indices, 98.27 and 98.77, respectively. At the same time, PSO was characterized by the lowest desaturase activity (74.34) among the tested oils. A high Δ9-desaturase (C18:1) index was evident in oils with a higher C18:1 level relative to C18:0, which is consistent with previous studies [4].

The desaturation pathways from LA to ALA and from C18:1 to LA were also assessed, as they may be useful for evaluating the desaturation potential of different oil plant phenotypes [25]. High ODR values indicate that the fatty acid biosynthetic pathway is efficient in forming LA from C18:1. The highest ODR ratios were noted for EPSO (80.29), SFO (82.00), and BPSO (82.75), which explains the large increase in LA content in these oils. For most of the oils, ODR values were found to be relatively high compared with LDR values, which may explain the increase in LA with a simultaneous decrease in ALA, as was also shown for some sesame oils [25], whereas relatively high values of both ODR and LDR indices in FSO, CSSO, MSO, and MTSO help explain the increased ALA content, as recently demonstrated for flaxseed oil by Belhoussaine et al. [9].

EFAs (LA and ALA), due to the lack of appropriate enzymes in the bodies of mammals, must be supplied with foods. Then, they are converted into a series of unsaturated n-3 fatty acids (as EPA and DHA) and n-6 fatty acids (as arachidonic acid) as a result of a series of successive elongation and desaturation processes [13]. In the case of dietary EFA deficiency, the body begins to produce n-9 fatty acids, including eicosatrienoic acid (20:3 n-9), as a result of Δ9 desaturation. However, as noted by Vessby et al. [42], changes in the type of dietary fats can also influence indices of fatty acid desaturase activity in plasma and skeletal muscle in human subjects and thereby change the fatty acids proportion in the body. Therefore, not only the dietary intake of fatty acids, but also the endogenous metabolism of fatty acids, e.g., by elongation and desaturation, can influence their effects in the body.

#### 2.1.3. Oxidizability Indexes

To predict the oxidizability of the tested oils, theoretical oxidizability indices such as the degree of unsaturation (UI), peroxidizability index (PI), oxidizability index (OI), as well as the oxidizability value (Cox) and oxidative susceptibility (OS), were calculated (Table 2).

The UI adds information on the overall degree of fatty acids’ unsaturation with respect to the unsaturation weight of each UFA but without distinguishing n-3 and n-6 of the fatty acids series. Although originally developed to reflect the nutritional aspect, it is also of great importance in establishing the oxidative stability of fats [4]. CSSO, FSO, and BSO exhibited the highest unsaturation level (UI = 203.52–171.97), followed by MTSO, BPSO, SFO, and EPSO (UI = 168.17–158.72). The lowest UI values were noted for PSO (132.03) and SO (130.40), which also revealed the lowest PUFA/SFA ratios.

The OI index is calculated based on the content of UFAs with 16 and 18 carbon atoms, whereas the PI index is used to assess the stability of PUFAs included in food products and their resistance to auto-oxidation [22]. Simultaneously, a higher PI value provides greater protection against coronary artery disease [43]. The same relationships observed for UI indicators were also found for OI among the analyzed oils, with CSSO and FSO showing the highest values (above 1) and PSO and SO the lowest (0.55 and 0.47, respectively). However, Czaplicki et al. [22] calculated a much lower oxidation index value for sea buckthorn oil, amounting to 0.16, which simultaneously was ca. 3.5 times lower than the value found in our study for SBO (0.57). The OI values for PSO and FSO were 0.55 and 1.08, respectively, which were identical (for PSO) or nearly identical (for FSO) to those reported by [21], indicating high oxidative stability for PSO and low stability for FSO. Similarly, higher OI values for FSO reported in other studies confirm this property [22]. A similar trend was noted for the PI indicator, which ranged from 46.86 in SO to 112.66 in CSSO. A comparable relationship between OI and PI in certain cold-pressed oils has also been noted by other researchers [21]. In general, oils with high PI values (CSSO, FSO, BSO) are characterized by higher levels of PUFAs, with high UFAs playing a key role in human health but being particularly vulnerable to oxidative damage. Hence, on the one hand, lipid peroxidation susceptibility increases with rising PI values, but on the other hand, a PI value greater than 80–90 is desirable from a health point of view [43,44].

The most commonly calculated indices used to determine the oxidative stability of the oils are Cox and OS, where Cox is typically considered for an assessment of the oil’s propensity to undergo auto-oxidation. Both values should be as low as possible to indicate that the fatty acids are less prone to oxidation, and thus, the oil is more stable [9]. The investigated oils showed moderate oxidative stability, with Cox values not exceeding 13. At the same time, the calculated Cox and OS values revealed notable differences among the tested oils. The lowest OS and Cox values were noted for SO (2017.08 and 5.12) and PSO (2466.17 and 5.82), indicating lower susceptibility to auto-oxidation in these oils. However, these values were still higher compared with those of argan oil [38]. The following oxidative stability index values were found for SBO (2565.6 and 6.13), BCSO (2646.59 and 6.24), and MSO (2709.09 and 6.40). Slightly higher OS and Cox values were calculated for EPSO (3204.9 and 7.47), SFO (3340.38 and 7.76), and BPSO (3399.98 and 7.88). The highest OS and Cox values were characterized for MTSO (3612.11 and 8.28), BSO (4149.25 and 9.29), FSO (5351.5 and 11.80), and CSSO (5598.47 and 12.28), mainly due to their high content of C18:3 fats. These Cox values are comparable to those reported for evening primrose and pumpkin seed oils but lower than those found for poppy, linseed, and black cumin oils by Symoniuk et al. [45] and for linseed oil by [9], whereas the Cox value calculated for CSSO was higher than the value provided for different camelina oils (9.5–10.1) by Ratusz et al. [30]. These discrepancies in the stability resulted from differences in the fatty acid composition of the oils.

Our research showed that all above-described indices exhibited comparable trends and revealed similar differences among the analyzed oils, as they were all determined based on FAME, considering the weighted degree of fatty acids unsaturation. Consequently, these indices are often used interchangeably by different researchers to theoretically determine the susceptibility of plant oils to oxidation [21,44]. Hence, due to the low values of all calculated theoretical oxidizability indices for SO and PSO, it appears that these oils may be suitable for human nutrition. Conversely, despite their high nutritional value, CSSO and FSO, with the highest PI and Cox values, are highly sensitive to oxidation, which may lead to the formation of unhealthy oxidation products in these oils. This highlights the imbalance between PUFA content and the level of protective antioxidants. This provides important insight for practical applications. To improve the oxidative stability of oils, additives such as natural or synthetic antioxidants are often added [17]. Recently, oil blends have gained increasing attention, with their compositions being carefully selected to achieve optimal nutritional properties while maintaining adequate oxidative stability [5,15,33].

### 2.2. Phenolic Compounds

In this study, a comprehensive quantitative analysis of twenty phenolic compounds was carried out in the investigated cold-pressed oils. The identified compounds covered a range of chemical classes, including flavonoids (apigenin, quercetin, luteolin, kaempferol), flavanols (catechin), glycosides (rutoside, vitexin), hydroxybenzoic and hydroxycinnamic acids (such as ferulic, caffeic, sinapic, and chlorogenic acids), and aromatic aldehydes (vanillic acid, vanillin). Individual compound concentrations, including standard deviations, are presented in Table 3.

Among all quantified phenolics, sinapic acid was the most dominant, reaching exceptionally high concentrations in BPSO (1838.5 mg/kg), BSO (1678.8 mg/kg), and MTSO (1630.0 mg/kg). The lowest content was recorded in SO (143.9 mg/kg), representing a more than twelvefold difference between the most and least concentrated samples. These results confirm sinapic acid’s central role in the phenolic composition of cold-pressed oils and support previous findings on its antioxidant potential and function in lipid peroxidation protection [31,46,47]. Chlorogenic acid levels were also highly variable, ranging from 58.3 mg/kg in BSO to 402.6 mg/kg in FSO. Notably high values were observed in EPSO (332.4 mg/kg), SBO (297.4 mg/kg), and BCSO (279.2 mg/kg). Oils rich in chlorogenic acid generally also contained high amounts of caffeic acid, with PSO (197.9 mg/kg), EPSO (194.8 mg/kg), and BCSO (189.4 mg/kg) showing the highest levels. The co-occurrence of these biosynthetically related phenolics suggests favorable extraction conditions and reflects their combined antioxidant activity [48]. Ferulic acid reached its peak in CSSO (292.8 mg/kg), followed by PSO, BCSO, and EPSO, where levels also exceeded 100 mg/kg. This compound is of particular technological interest due to its potential synergistic role with tocopherols in improving oxidative stability [49].

Among the hydroxybenzoic acids, 4-hydroxybenzoic acid was most abundant in CSSO (196.2 mg/kg), MTSO (168.7 mg/kg), and BPSO (136.0 mg/kg). Other acids in this group, including gallic and protocatechuic acids, exhibited more consistent levels across oils, typically ranging between 16 and 68 mg/kg, supporting their chemical persistence in lipid matrices [47]. Within the flavonoid class, quercetin was the most prevalent, particularly in MTSO (209.9 mg/kg) and BSO (167.4 mg/kg), while SO and EPSO exhibited the lowest concentrations (24.5 and 65.6 mg/kg, respectively). Apigenin exceeded 100 mg/kg in EPSO, SFO, and BSO, but dropped below 40 mg/kg in MSO and SO. Luteolin showed a more even distribution, with the highest levels in BSO (66.1 mg/kg), EPSO (62.9 mg/kg), and SO (58.6 mg/kg). Kaempferol was most abundant in MSO (49.8 mg/kg) and showed notably lower levels in MTSO and BSO. Naringenin was primarily detected in BCSO (51.2 mg/kg) and PSO (48.9 mg/kg), while MSO and SO again showed the lowest concentrations. The only flavanol detected, catechin, showed the highest content in BCSO (75.0 mg/kg), FSO (65.8 mg/kg), and BPSO (46.7 mg/kg), while remaining below 16 mg/kg in EPSO, PSO, and SO. Glycosides, such as rutoside and vitexin, occurred in moderate concentrations across all oils. Vitexin levels were particularly elevated in BCSO (51.1 mg/kg) and remained consistently above 30 mg/kg in most samples, while rutin levels generally ranged from 12 to 18 mg/kg. Though minor in quantity, these glycosides may still influence oil stability due to their potential conversion to active aglycones [50]. Phenolic aldehydes, including vanillin and vanillic acid, were found in the lowest concentrations among all quantified phenolics. Vanillin appeared in trace amounts only, peaking in CSSO (4.3 mg/kg) and averaging below 1 mg/kg in most oils. In contrast, vanillic acid reached 33.2 mg/kg in FSO and was relatively stable across the samples, suggesting better chemical resilience. Despite their low abundance, these compounds may contribute to the sensory and oxidative profiles of the oils [48].

The total phenolic content (TPC), calculated as the sum of all quantified compounds, was highest in BPSO (2884.9 mg/kg), MTSO (2860.2 mg/kg), and BSO (2761.0 mg/kg), reflecting their richness in bioactive and antioxidant agents. On the other hand, the lowest TPC values were recorded in SO (937.5 mg/kg), MSO (941.6 mg/kg), and SFO (1002.5 mg/kg). This wide variation supports previous findings that phenolic content is strongly influenced by seed variety, oil matrix composition, and extraction efficiency [27].

### 2.3. Tocopherols

Among the studied oils, SBO and FSO contained the highest total tocopherols contents, at 109.88 and 84.16 mg/100 g, respectively (Table 4), with γ-tocopherol (γ-T) clearly dominating in these oils, at 75.02 and 79.93 mg/100 g in SBO and FSO, respectively. These γ-T levels were similar to, or slightly higher than, those reported in previous studies [31,38,51]. However, significantly lower values of γ-T in FSO, with a mean of 12.4 mg/100 g, have also been reported [52]. At the same time, FSO characterized only a few percentages of α-tocopherol (α-T) (4.23 mg/100 g), which is consistent with data reported by other authors for this oil [31]. SFO and SO showed comparable total tocopherol contents, 65.93 and 64.85 mg/100 g, respectively, with very similar compositions of α-T and γ-T (27.48 and 26.07 mg/100 g for α-T and 36.74 and 38.36 mg/100 g for γ-T, respectively).

There were no significant differences between BPSO, CSSO, and MTSO in total tocopherols contents; however, notable variations were observed in the amounts of individual tocopherols. BPSO was characterized by nearly equal levels of α-T and γ-T, whereas MTSO contained 1.7 times more γ-T than α-T, which is opposite to some previous reports [31]. In CSSO, the highest level of α-T and simultaneously the lowest level of γ-T were noted, 44.87 and 10.37 mg/100 g, respectively, which is opposite to [31], where α-T was not detected in CSSO. PSO exhibited a comparable amount of γ-T and a slightly lower level of α-T (11.60 and 35.03 mg/100 g, respectively). At the same time, it was the only oil that contained a significant amount of δ-tocopherol (δ-T), at 13.63 mg/100 g. However, some of the literature sources show a significantly higher γ-T content in PSO along with similar levels of α-T and δ-T [31].

BSO, MSO, and BCSO had similar α-T contents, ranging from 16.79 to 19.23 mg/100 g. Among these oils, in terms of γ-T, BSO (36.61 mg/100 g) showed the highest content, followed by BCSO (25.63 mg/100 g) and MSO (17.85 mg/100 g). This means that MSO was also characterized by the lowest total tocopherol content (37.88 mg/100 g). BCSO was the only oil in which a few percentages of β-tocopherol (β-T) were determined (3.34 mg/100 g), although a higher β-T level in BSCO was found in [53] and a lower one in [24]. EPSO, after FSO, contained the lowest α-T amount (10.63 mg/100 g) and had a γ-T content (32.77 mg/100 g) comparable to MTSO, BSO, SO, and SFO.

The differences observed in tocopherol composition and concentration between the present results and those in the literature can be explained by genetic and agronomic factors, quality characteristics of seeds, and technological factors [54].

The tested oils were characterized by high levels of total tocopherols, ranging from 37.87 in MSO to 109.88 mg/100 g in SBO, where α-T and/or γ-T were the dominant forms, concerning previous reports [22]. β-T and δ-tocopherols (δ-T) were either not detected or found at low levels (below 3.34 mg/100 g) in the analyzed oils with the exception of PSO, which contained 13.63 mg/100 g of δ-T, the secondary tocopherol after α-T in this oil.

Although α-T is appreciated by consumers as a beneficial component of oil, γ-T, when present at elevated levels, contributes significantly to the oil’s antioxidant capacity [55]. Among the analyzed oils, the highest levels of γ-T were detected in FSO and SBO, which may influence their oxidative stability. It is also known that antioxidant properties depend, among other factors, on the concentration of bioactive compounds. Studies have demonstrated that α-T exhibits its highest antioxidant activity in vegetable oils at a concentration of 100 mg/kg, whereas concentrations above 250 mg/kg may lead to a slight pro-oxidant effect. On the contrary, γ-T shows the most potent antioxidant properties at concentrations between 250 and 500 mg/kg [31]. Furthermore, during prolonged storage of oils, a significantly greater reduction in α-T concentration has been observed compared with γ-T, which strongly correlates with the formation of lipid hydroperoxides [31]. In contrast, δ-T has shown stronger antiradical properties than the γ-homologs in some studies [56]. Although tocopherols are known as the main lipid-phase antioxidants, in some cases, other bioactive compounds can also play a significant role in antioxidant activity. For instance, in pumpkin seed oil, 59% of the antioxidant capacity was attributed to polar phenolics, while only 41% was due to tocopherols [55].

### 2.4. Pigments

Pigments such as chlorophylls and carotenoids in cold-pressed oils originate from the plant seeds and are not removed during the cold pressing. Their content is influenced by many factors, including plant cultivation, seed maturity, and the type of pressing method used [27,51]. As reported, the chlorophyll level in edible oils should not exceed 50 mg/kg due to its pro-oxidative effects, which negatively impact the oxidative stability of the oil and accelerate rancidity, thereby shortening its shelf life [45]. However, in the absence of light, chlorophylls can act as antioxidants [9,31]. In contrast, carotenoid pigments function as antioxidants by protecting triacylglycerols, unsaturated lipids, membranes, and phenolic quinones from photo-oxidative processes. They are also known for their cancer-preventive properties [51].

Chlorophyll and carotenoid pigments were determined spectrophotometrically and expressed as chlorophyll *a* and β-carotene in mg per 100 g of oil (Table 4). Chlorophyll content ranged from 0.02 mg/100 g (in SO and SFO) to 0.89 mg/100 g (in BCSO), which is below the acceptable level in edible oils. The exception was PSO (21.14 mg/100 g), mainly due to the presence of a high content of pheophytins, the main dye in this oil [31]. Similarly low chlorophyll levels have previously been published for camelina oils, varying from 1.02 to 2.18 mg/kg [30]. In contrary, higher chlorophyll levels were noted in [52]. Although most of the analyzed oils have chlorophyll levels below the tolerable limit (< 0.9 mg/100 g), this does not exclude the possibility of photochemical oxidation being induced at such concentrations [57].

Concerning carotenoid pigments, among all the analyzed oils, PSO (8.96 mg/100 g) contained the second-highest level of carotenoids after SBO (229.22 mg/100 g), which showed the highest one. The carotenoid levels in the remaining oils ranged from 0.10 to 1.35 mg/100 g, which were lower in CSSO, EPSO, MTSO, and BCSO than the values reported in the literature [45]. However, some studies have shown similar or even lower carotenoids levels in these oils [9,22,27,51]. As previous results on the effect of β-carotene on the light stability of soybean oil have shown, the presence of this pigment at concentrations between 5 and 20 mg/kg of oil provides a protective effect against oxidative damage induced by light [58]. Regarding these results, only the carotenoid content of FSO, BCSO, and MSO could be considered sufficient to provide some protection against photo-oxidative degradation.

A source of discrepancy in the spectrophotometric measurement of pigments in unrefined oils may be the calibration of the method for only a specific type of pigment, most often chlorophyll *a* or pheophytin *a*, and β-carotene or lutein, for chlorophylls and carotenoid pigments, respectively. Although their absorption maxima occur at the same wavelength, the molar absorption coefficients differ significantly, additionally depending on the solvent used. This may explain the different results obtained by diverse researchers employing different methods.

Previous research has demonstrated that in unrefined oils, the ratio of carotenoids to chlorophylls is variable [59]. In our study, most of the tested oils contained higher carotenoid levels than chlorophyll (ranging from 0.10 to 229.22 mg/100 g), except for BCSO, PSO, and BSO, where chlorophylls predominated. Earlier studies also confirmed that chlorophylls are the most abundant pigments in these oils [45]. As shown in Table 4, the richest source of carotenoids, excluding SBO, was PSO (8.96 mg/100 g), and the poorest one was SO (0.10 mg/100 g). A similar trend among unrefined oils was also observed previously [59]. SBO was characterized by the highest carotenoid content, reaching a level similar to that noted in the literature (approx. 200 mg/100 g) [22]. Such a high carotenoid content provides regenerative and antiwrinkle properties of this oil, with β-carotene identified as the main carotenoid responsible for sea buckthorn oil’s characteristic orange-red color [60].

### 2.5. Specific Extinction Coefficients (K_232_ and K_268_)

Lipid oxidation represents the primary mechanism responsible for quality degradation in fat-containing foods. The oxidative stability of oils is primarily influenced by their fatty acid composition and the presence of antioxidants. The primary changes occurring in stored oils result from the presence of double bonds in fatty acids, with susceptibility to oxidation increasing rapidly as the number of double bonds per molecule rises. Consequently, PUFAs are considerably more susceptible to oxidative degradation than MUFAs or SFAs [61]. The amounts of conjugated products formed from PUFAs, described as conjugated dienes, are proportional to the content of hydroxyl peroxide compounds [30]. Secondary oxidation products, such as conjugated trienes, were also determined at the beginning of the heating process and within a time of storage of 21 days at 60 °C. The results were expressed as K_232_ and K_268_ extinction coefficients, respectively. The obtained results for SO, MSO, SBO, BPSO, BSO, and SFO oils are given in Table S1, while data for the remaining oils are presented in our earlier study [20].

Significant differences in conjugated products between the oils were noted at the start point of the heating test. The analyzed oils showed K_232_ values ranging from 1.60 to 12.30, while K_268_ values were much lower, ranging from 0.03 to 2.70. The highest concentrations of conjugated dienes were noted in SFO and SBO, at 10.55 and 12.30, respectively, indicating a significant accumulation of oxidation products. The highest K_268_ value was detected for PSO (2.70), simultaneously with a significant value of conjugated trienes (7.50). Among the analyzed oils, CSSO exhibited the lowest K_232_ (1.60) and K_268_ (0.03) extinction values. Similar results were achieved for CSSO by Ratusz et al. [30]. Although there are no standardized limit values for K_232_ and K_268_ for cold-pressed oils, some researchers have discussed their results referring to the EU Commission Regulation 2568/91 for extra virgin olive oil [15,30]. According to this regulation, K_232_ and K_268_ values should not exceed 2.50 and 0.22, respectively. Following these guidelines, only FSO and CSSO met the given criteria, while BSO was at the threshold.

The reason for the higher K values observed in other studied oils, indicating a significant content of oxidation products, may resulted from technological processes or oil storage conditions. However, it also may be attributed to the co-occurrence of some compounds absorbing in the same wavelength range, such as carotenoids present in pumpkin seeds [62]. Similarly, values exceeding the aforementioned norms for several cold-pressed oils, including BSCO, PO, and EPSO, have been noted by other researchers [15,31].

During storage, the level of both primary and secondary oxidation products increased at diverse rates depending on the oil, highlighting variations in oxidative stability and susceptibility to degradation. In all oils, a significant increase in these parameters was observed after about 10–14 days of the test. After 18 days, in some oils (MSO, SBO, SFO, EPSO) the K_232_ value began to decrease, which may be connected with the peroxide concentration reaching its maximum and next decomposing into secondary oxidation products [5]. Hence, based on the obtained K values, to better visualize the oils’ susceptibility to oxidation, the growth rates of K_232_ and K_268_ were calculated after 10 days, 14 days, and at the endpoint—either after 18 days (for MSO, SBO, SFO, and EPSO) or after 21 days (for the remaining oils). These results are summarized in Table 5.

At the initial stage of storage, the growth rates of the K parameters ranged from 1.04 to 6.80 for K_232_ and from 0.82 to 24.40 for K_268_, with BPSO and CSSO showing the fastest growth rates of K_232_ and K_268_, respectively. A significant growth rate of secondary oxidation products was also observed for EPSO (18.58). At the same time, CSSO, together with MSO, exhibited a significant higher rate of K_232_ growth compared with the other oils, while the lowest increases in both primary and secondary oxidation products were noted for BCSO and PSO. The high oxidative stability of BCSO and PSO, in comparison with some other cold-pressed oils, has also been reported by other studies [5,15].

After 14 days, the growth rate of K_232_ increased comparably for most oils, enlarged 1.3 (for CSSO and SO) to 2.14 (for BSO) times. Exception were BCSO and PSO, where no significant differences were observed, and MSO, where the K_232_ growth rate slightly decreased. By the end of the test, the fastest growth of oxidation product occurred in FSO (growth rate 33.17), achieving an 8- and 4.25-fold increase compared with 10 and 14 days, respectively. Similarly low oxidative stability during storage for FSO has also been observed by other researchers [31]. In the case of FSO, the oxidation rate was particularly high despite its significant levels of γ-T (79.93 mg/100 g) and polyphenols (2293 mg/kg). This suggests a possible threshold beyond which antioxidant compounds are insufficient to prevent lipid peroxidation, or which counteracts the effects of the presence of trace pro-oxidant agents such as metal ions. However, further studies are warranted to confirm this hypothesis.

More significant variances occurred in the K_268_ growth rate, which increased most notably after 14 days in BPSO (by 3.8 times). The largest percentage change in conjugated products during storage of poppy seed oil was also reported by [23]. By the end of the storage period, the K_268_ growth rate increased most significantly for EPSO and CSSO, rising rapidly by about 4–8 times compared with the initial stage of storage, indicating a sharp increase in the oils’ degradation.

Significant differences were observed between the oils at both the first and subsequent stages of oxidation. The lowest degradation rates were observed for PSO and BCSO throughout the entire storage period. A slow increase in K values was also noted for SO, but only in the initial storage period. The fastest oxidation process was recorded in FSO, confirming previous reports [15,21,22,38].

### 2.6. Correlations

To investigate the influence of the analyzed FAME parameters and phytochemicals (fatty acid composition and the content of chlorophylls, carotenoids, phenolics, and tocopherols) on the oxidative stability of the oils, a statistical analysis was performed. The results of the correlation analysis between selected quality variables and oxidative stability, determined based on the presence of conjugated dienes and trienes (K_232_, K_268_, and their growth rates) as well as theoretical Cox values, are presented in Table 6.

In our previous study, it was found that the K_232_ and K_268_ values for MTSO, EPSO, FSO, CSSO, BTSO, and PSO mostly reflected the susceptibility of these oils to oxidation related to their PUFA contents [20]. Similarly, when considering all oils analyzed in the present study, a comparable relationship was found between PUFA content and K values at the end of the thermostatic tests, with a significant (*p* ≤ 0.05) positive correlation (r = 0.66 and r = 0.71 for K_323_ and K_268_, respectively). Moreover, a strong positive correlation (r = 0.91) between K_232_ and K_268_ confirmed a close connection between the decomposition of primary into secondary oxidation products [5]. Additionally, the presented relationship results revealed a significant positive correlation between K_268_ and C18:2 content (r = 0.59). However, when considering the growth rate of K values, no statistically significant correlations were obtained between them and either the fatty acid groups (PUFA, MUFA, or SFA) or individual fatty acid contents. The exception was C18:3, as one of the most susceptible fatty acids to oxidation, which had the greatest impact on the growth rate of primary oxidation products at the end of the test (r = 0.67). Symoniuk et al. [15,45] also noted no significant effect of the overall content of individual fatty acid groups on the oxidative stability of some cold-pressed oils determined by the Rancimat method (oil oxidation at 100 °C), simultaneously with a significant impact of C18:3 on the oxidative stability of the oils (r = −0.64 and r = −0.54). Similarly, Ratusz et al. [30] found no significant correlations for camelina oils using both the Rancimat and Pressure Differential Scanning Calorimetry (PDSC) methods. On the other hand, Maszewska et al. [18] indicated that oxidative changes in some cold-pressed oils were of a similar nature and comparable in both the Rancimat and thermostatic test, as confirmed by the high correlation between results obtained from each method. The correlation results in our study, compared with the above-mentioned outcomes, seem to confirm this relationship.

As indicated earlier, the relationship between an oil’s susceptibility to oxidation and its potential oxidizability is rarely noted, since oil is a complex mixture of compounds. The oxidative stability of oils is also influenced by the presence of antioxidants. Relationships between oxidative stability and antioxidant content have been found significantly more often [15,30,45]. However, in our study, merely significant negative correlations (*p* ≤ 0.05) were identified between *α*-T and K_268_ growth rates (r = (−0.72)–(−0.63)). Based on the data given in Table 6, it can be concluded that the oxidative stability of the tested oils does not depend directly on any individual component. Instead, it has a complex nature influenced by many parameters. At first, vegetable oils are a rich source of various substances with antioxidant activity. Moreover, their composition and concentration can also impact their synergistic or antagonistic activity as well as lipophilic or hydrophilic properties [5,22,31]. Hence, high correlations can be found between individual bioactive components of the oils [22,27,31], although in our results, only carotenoids, which are considered to be the main lipid antioxidants, were highly positively correlated with total tocopherols content (r = 0.79). Synergistic interactions, including those between tocopherols and β-carotene, have been demonstrated in palm oil and in sea buckthorn oil blends [22].

**Figure 1:**
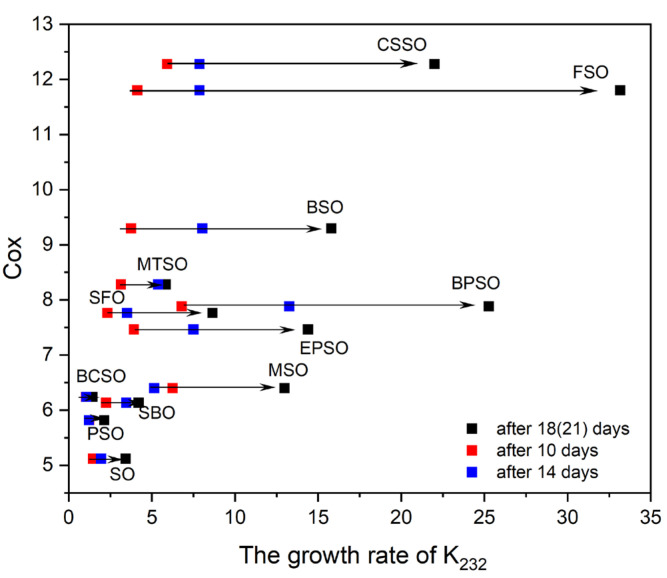
The relations between Cox and the grow rates of K232 after 10, 14, and 18 (or 21) days.

The theoretical Cox values and their relationship to the growth rates of K_232_ after 10, 14, and 18 (or 21) days of the thermostatic test for the investigated oils, presented in Figure 1, confirm the complexity of the observed processes. The lowest Cox index, obtained for SO (Section 2.1.3), followed by PSO, indicated higher oxidative stability for these oils. However, BCSO exhibited the slowest K_232_ increase. On the other hand, the highest Cox value was calculated for CSSO, although FSO, rich in bioactive compounds, exhibited the lowest oxidative stability, similar to BPSO, which had a moderate Cox value. Therefore, the Cox index was positively correlated with the growth rates of K_232_ after 14 days (r = 0.59) and more significantly with the K_232_ growth rate at the end of the test (r = 0.79). This finding is in contrast with other results where no correspondence among Cox and the oxidation induction time, as measured by the Rancimat method, was observed [30,45]. However, the literature also provides data showing a high correlation between the Cox index and the oxidative stability established with the PDSC method [45]. Moreover, some studies showed that FAME could be a predictor for oil oxidation stability at an early stage of oil oxidation but not in later steps [21]. This is opposite to our findings, where correlations improved over time. In our case, the best correlations were observed at the end of the test, where the fatty acids composition became the dominant factor rather than the bioactive pro- or antioxidants’ ingredients.

**Figure 2:**
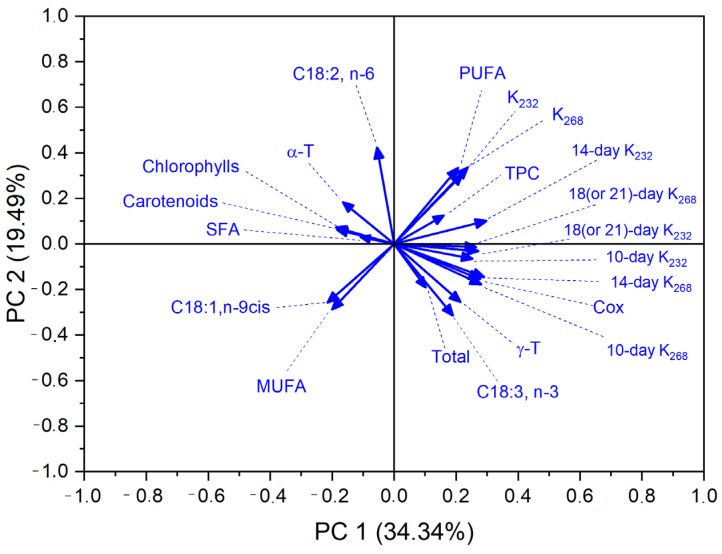
Principal component analysis (PCA) based on quality features of analyzed oils.

To further investigate the relationships between fatty acid composition, the content of bioactive compounds, and the oxidative stability of the analyzed oils, a principal component analysis (PCA) was performed. The results are presented both graphically (Figure 2) and in tabular form (Table 7). The PCA enabled dimensionality reduction and the identification of variables with the greatest influence on sample variability and susceptibility to oxidation.

The first two principal components (PC1 and PC2) explained 53.83% of the total variance (34,34% and 19,49%, respectively), indicating a strong explanatory value of the model. Although the first two principal components accounted for only 53.83% of the total variance, PCA was used primarily as an exploratory tool to visualize clustering patterns and general relationships among samples and quality indices. Additional components, while explaining more variance, did not significantly improve the interpretability of the data. As shown in Table 7, the highest positive loadings for PC1 were observed for oxidation indicators (K_232_, K_268_), their growth over time (especially after 10 and 14 days), PUFA content, and the Cox value. The strong positive association among these variables confirms their collective contribution to oxidation processes and their mutual interdependence. Particularly high loadings were noted for 14-day K_232_ (0.2978) and the Cox value (0.2772), emphasizing their importance in distinguishing the oxidative stability of oils. C18:3, known for its high oxidative susceptibility, also showed relatively high positive loadings on both PC1 and PC2, further supporting its central role in the initiation of primary oxidation reactions. This suggests that C18:3 content may serve as a meaningful predictor of early oxidative changes in oils. In contrast, antioxidant-related variables such as α- and γ-T, carotenoids, and chlorophylls had lower loading values and vectors oriented in the opposite direction to the oxidation indicators (Figure 2), suggesting their potential protective effect, particularly against the formation of secondary oxidation products (K_268_). Notably, α-T showed a strong inverse relationship with the growth rate of K_268_, which is consistent with earlier correlation results (r = −0.72 to −0.63).

Furthermore, both the PCA plot and the tabular data highlight the interrelations among bioactive components. A strong positive correlation was observed between carotenoids and total tocopherol content, suggesting potential synergistic antioxidant effects. Overall, the PCA results confirm the complex nature of oil oxidative stability, determined by a combination of pro-oxidant factors (e.g., PUFAs) and antioxidant compounds, along with their interactions.

## 3. Materials and Methods

### 3.1. Materials (Oil Samples)

The research material consisted of twelve cold-pressed vegetable oils derived from evening primrose seeds (*Oenothera paradoxa*)—EPSO; milk thistle seeds (*Silybum marianum*)—MTSO; pumpkin seeds (*Cucurbita oleo*)—PSO; flaxseeds (*Linum usitatissimum* L.)—FSO; camelina sativa seeds (*Camelina silvestris*)—CSSO; black cumin seeds (*Nigella sativa*)—BCSO; blue poppy seeds (*Papaver* L.)—BPSO; safflower seeds (*Carthamus tinctorius* L.)—SFO; borage seeds (*Borago officinalis*)—BSO; sea buckthorn seeds (*Hippophae rhamnoides* L.)—SBO; and sesame seeds (*Sesamum indicum* L.)—SO. These oil samples were commercially purchased from Polish suppliers (SemCo Sp. z o.o. Sp.k., Szamotuły, Poland and NaturOil, Kościan, Poland) and stored in amber glass bottles at 4 °C until analysis. All analyses were conducted within three weeks of opening the bottles. To evaluate the oxidative stability of the selected oils, an accelerated storage test was performed under elevated temperature conditions (Schaal oven test). The samples were stored in an oven at 60 °C under ambient air conditions to mimic the oxidative processes that occur over time and to speed up oxidation reactions. Under these conditions, one day at 60 °C corresponds to approximately one month of storage at room temperature [63]. Samples were taken for analysis after 3, 6, 10, 14, 18, and 21 days of storage.

### 3.2. Chemicals

Sodium methoxide and hexane (purity 99.9%), used for methylation in the FAME analysis, were obtained from Sigma-Aldrich, St. Louis, MO, USA. Pentane (C_5_H_12_), used for lipid extraction after neutralization, was supplied by Sigma-Aldrich, USA. *n*-Hexane (C_6_H_14_), used in the methylation process and lipid extraction, was also sourced from Sigma-Aldrich, USA. Sulfuric acid (H_2_SO_4_), used in the methanol/sulfuric acid mixture for methylation, was provided by Chempur, Piekary Śląskie, Poland. Isooctane (2,2,4-trimethylpentane) was obtained from POCH, Lublin, Poland. These high-purity solvents were essential for the accurate extraction, preparation, and analysis of the oils.

### 3.3. Fatty Acid Composition

#### 3.3.1. Fatty Acid Analysis

The fatty acids profile was determined in the analyzed oils by gas chromatograph. The method used was the AOCS (2005) method [64]. Sample preparation and chromatographic separation conditions are described in detail in a previous publication [37].

#### 3.3.2. Qualitative, Nutritional, and Metabolic Fatty Acid Indexes

The fatty acid compositions of the tested oils were used to calculate several qualitative indices. These included the content of saturated (SFAs), unsaturated (UFAs), monounsaturated (MUFAs), and polyunsaturated (PUFAs) fatty acids as well as the PUFA/SFA ratio and the n-6/n-3 ratio. Additionally, the following indices were calculated based on the fatty acid content of the oils:

The atherogenic index (AI) and the thrombogenic index (TI), following Ulbricht and Southgate [28]:

AI = [C12:0 + (4 · C14:0) + C16:0]/(ΣUFA)

TI = (C14:0 + C16:0 + C18:0)/[(0.5 · MUFA) + (0.5 · Σn-6 PUFA) + (3 · Σn-3 PUFA) + (Σn-3 PUFA/Σn-6 PUFA)].

The ratio of hypocholesterolemic to hypercholesterolemic fatty acids (h/H) based on the Santos, Bessa, and Santos equation [40]:

h/H = (C18:1 + ΣPUFA)/(C12:0 + C14:0 + C16:0).

The unsaturation index (UI), following the equation given in [4]:

UI = (%monoenoic) + (2 · %dienoic) + (3 · %trienoic) + (4 · %tetraenoic) + (5 · %pentaenoic) + (6 · %hesaenoic).

Peroxidizability index (PI), calculated as follows:

PI = (monoenoic acid · 0.025) + (dienoic acid · 1) + (trienoic acid · 2) + (tetraenoic acid · 4) + (pentanoic acid · 6) + (hexanoic acid · 8).

The oxidizability value (Cox) by applying the formula used in [9]:

Cox = (C18:1 + (10.3 · C18:2) + (21.6 · C18:3))/100.

Additionally, the oxidative stability (OS) and oxidizability index (OI) were determined using the formulas provided by Bielecka et al. [21] and Plaha et al. [44]:

OS = MUFA + (45 · C18:2) + (100 · C18:3)

OI = (0.02 · (C16:1 + C18:1) + 1 · C18:2 + 2 · C18:3)/100.

Finally, using an enzymatic approach called the “products/substrate ratio,” a variety of indices were used for metabolic indicators in order to quantify the activity of desaturases and elongase [4,9]:

Elongase = (C18:0/C16:0) · 100

Desaturase (Δ9-desaturase (C18:1)) = (C18:1/(C18:0 + C18:1)) · 100

Oleic desaturation (OLD) = ((C18:2 + C18:3)/(C18:1 + C18:2 + C18:3)) · 100

Linoleic desaturation (LDR) = (C18:3/(C18:2 + C18:3)) · 100.

### 3.4. Phytochemical Composition

#### 3.4.1. Tocopherol Content

Tocopherol composition was analyzed according to the method described in [65]. A 1 mL oil sample was subjected to the saponification process: oil and 0.5 g of pyrogallol were added to a round-bottomed flask, and 20 mL of anhydrous ethyl alcohol and 2 mL of 60% KOH were added. After heating for 30 min at the boiling point of the solvent, 50 mL of 1% NaCl was added to the samples, the samples were thoroughly cooled. Then 50 mL of *n*-hexane with 10% ethyl acetate was added. The tightly closed flasks were shaken (at 300 rpm) for 30 min. Then, about 2 mL of saturated NaCl solution was added. After 15 min, an appropriate amount was taken from the upper layer (unsaponifiable substances) for injection into a liquid chromatograph (UPLC). The recovery of tocopherol standards saponified by this method is 99.9%, as reported in the official standards (PN-EN 12822:2002; PN-EN ISO 9936:2006) and in the study by Ryynänen et al. (2004) [66,67,68]. Tocopherols were identified qualitatively and quantitatively on a UPLC liquid chromatograph (an Acquity H class UPLC system, Waters, Milford, MA, USA) in a system consisting of a Waters 600 pump, Acquity UPLC^®^ BEH C18 column (100 mm × 2.1 mm, particle size 1.7 μm) (Waters, Wexford, Ireland) and a fluorometric detector. The mobile phase was a mixture of *n*-hexane with 1,4-dioxane (97:3 *v*/*v*). The flow rate was 1.5 mL·min^−1^. The fluorometric detector (Waters 474 Asc. Milford, MA, USA) was operated at excitation λ = 290 nm and emission λ = 330 nm. The concentration of individual tocopherol homologs was calculated from the previously prepared calibration curve.

#### 3.4.2. Chlorophyll and Carotenoid Pigments

Chlorophyll and carotenoid pigment contents were analyzed using the spectrophotometric method described in [9] based on specific extinction coefficients. Oil solutions in hexane at a concentration of 1% (*m*/*v*) were centrifuged using a 5417R centrifuge (Eppendorf, Hamburg, Germany) at 1600 rpm for 10 min. Absorbance was measured with a Shimadzu UV-1201 spectrophotometer (Kyoto, Japan) at 450 nm and 660 nm for carotenoid and chlorophyll pigments, respectively. The specific extinction coefficients used were 1000 for β-carotene and 2500 for chlorophyll *a*. Pigment contents were calculated as follows:

[Chlorophylls] = A_660_ · 10^6^/(1000 · 100 · d)

[Carotenoids] = A_450_ · 10^6^/(2500 · 100 · d)

where A is the absorbance and d corresponds to the optical path length of the cell (1 cm).

The result of measurement was expressed in mg of chlorophyll per 100 g of oil. Carotenoid content was expressed as β-carotene in mg per 100 g of oil.

#### 3.4.3. Individual Phenolic Compounds (HPLC)

Phenolic compounds in samples were analyzed after alkaline and acidic hydrolysis [69]. Analysis was performed using an Aquity H class UPLC system equipped with a Waters Acquity PDA detector (Waters, USA). Chromatographic separation was performed on a Acquity UPLC^®^ BEH C18 column (100 mm × 2.1 mm, particle size 1.7 μm) (Waters, Ireland). The elution was carried out by gradient following mobile phase composition: A: acetonitrile with 0.1% formic acid; B: 1% aqueous formic acid mixture (pH = 2). Concentrations of phenolic compounds were determined using an internal standard at wavelengths λ = 320 nm and 280 nm. Compounds were identified based on a comparison of retention time of the analyzed peak with the retention time of the standard and by adding a specific amount of the standard to the analyzed samples and a repeated analysis. Detection level was 1 μg/g. Retention times of assayed compounds were as follows: kampferol 6.11 min, gallic acid 8.85 min, vanillic 9.71 min, luteolin 11.89 min, protocatechuic acid 12.23 min, vanillin acid 14.19 min, apigenin 16.43 min, catechin 18.09 min, 4-hydroxybenzoic acid 19.46 min, chlorogenic acid 21.56 min, caffeic acid 26.19 min, syringic acid 28.05 min, naringenin 31.22 min, vitexin 35.41 min, rutin 38.11 min, quercetin 39.58 min, p-coumaric acid 40.20 min, ferulic acid 46.20 min, synapic acid 48.00 min, and t-cinnamic acid 52.40 min, respectively. Recovery rates for the analyzed phenolic compounds were as follows: kampferol 86 ± 5.3%, gallic acid 92 ± 4.4%, vanillic 79 ± 8.5%, luteolin 96 ± 2.7%, protocatechuic acid 90 ± 4.8%, vanillin acid 88 ± 5.1%, apigenin 93 ± 3.8%, catechin 89 ± 5.7%, 4-hydroxybenzoic acid 96 ± 3.78%, chlorogenic acid 92 ± 2.8%, caffeic acid 86 ± 6.7%, syringic acid 94 ± 3.9%, naringenin 88 ± 4.8%, vitexin 95 ± 3.8%, rutin 93 ± 4.9%, quercetin 97 ± 1.9%, p-coumaric acid 89 ± 3.6%, ferulic acid 91 ± 4.9%, synapic acid 94 ± 5.1%, and t-cinnamic acid 97 ± 2.9%.

### 3.5. UV–VisSpecific Absorbance Coefficients (K_232_, K_268_)

The oxidative stability of twelve oils was evaluated by calculating the specific absorbance coefficients (K_232_, K_268_), following the method described in our previous study [20]. For all oils, the K coefficients were determined at the start point of the test and after 3, 6, 10, 14, 18, and 21 days of storage. Based on the obtained values, the growth rates of K_232_ and K_268_ were calculated after 10 and 14 days, as well as after the day when the maximum K value was reached. This point was established based on the inflection point of the peroxide values for each oil. For oils that showed earlier inflection points, the value was set as 18 days, while for the remaining oils, it was considered as the final day of the Schaal test (21 days).

### 3.6. Statistical Analysis

The measurements were performed in triplicate (unless otherwise stated in the method description). The values of the analyzed variables are presented as mean ± standard deviation (SD). The obtained results were statistically processed using OriginPro Software for Windows, Version 2023b (OriginLab Corporation, Northampton, MA, USA). The significance of differences between groups was determined based on one-way analysis of variance (ANOVA) and Tukey’s post hoc test (*p* < 0.05). Differences between results for respective oils marked with the same letter in the same row are statistically insignificant (*p* < 0.05). Relationships between variables were analyzed using Pearson’s correlation coefficient (r) and principal component analysis (PCA) in OriginPro, with values of *p* < 0,05 considered statistically significant.

## 4. Conclusions

Due to the species diversity of the plants from which the oils were pressed, the fatty acids profile of the oils varied. However, all oils showed a predominance of UFAs over SFAs. The results of the nutritional indexes showed that the PSO slightly exceeds established recommendations, but only to a negligible extent. The PI index confirmed the high nutritional potential and protective effect, in terms of CHV, of these oils: FSO and CSSO. However, both FSO and CSSO were characterized by low oxidative stability, as confirmed by the OI, Cox, OS, and specific extinction coefficients values. In addition to fatty acids, other bioactive compounds were analyzed in the oils. The highest total polyphenol content was found in BPSO, which also had the highest synapic acid content. Comparing the content of tocopherols, tocopherols were highest in SBO oil. The most carotenoids were also found in SBO. All these compounds contribute to the antioxidant potential of the oils. It is different for chlorophylls, which have a pro-oxidant effect. A high content of chlorophylls was found in PSO. The ratio of SFAs and UFAs, as well as the presence of antioxidant and pro-oxidant compounds, affect the oxidative stability of oils. Based on the correlation performed, it was found that C18:2 and C18:3 are the main factors influencing fat oxidation. The antioxidant effect in oils can be attributed to a number of bioactive components, of which α-T showed the greatest correlation.

## Figures and Tables

**Table 1:** The fatty acids profile (FAME) of investigated oils.

Fatty Acid [%]	BPSO	BSO	MSO	SO	SFO	SBO
C14:0	nd	nd	nd	nd	nd	0.07 ± 0.00 ^a^
C16:0	9.26 ± 0.21 ^b^	10.57 ± 0.32 ^a^	3.48 ± 0.14 ^e^	10.57 ± 0.19 ^a^	7.39 ± 0.21 ^c^	6.08 ± 0.24 ^d^
C16:1	nd	nd	0.11 ± 0.00 ^b^	0.18 ± 0.00 ^a^	nd	0.08 ± 0.00 ^c^
C18:0	1.88 ± 0.12 ^e^	3.71 ± 0.15 ^b^	2.11 ± 0.10 ^d^	4.45 ± 0.17 ^a^	3.29 ± 0.16 ^b^	2.97 ± 0.10 ^c^
C18:1	15.33 ± 0.59 ^e^	17.09 ± 0.88 ^d^	48.88 ± 0.52 ^a^	38.75 ± 0.62 ^b^	16.08 ± 0.33 ^d^	33.83 ± 0.42 ^c^
C18:2	72.17 ± 0.45	37.52 ± 0.33	29.08 ± 0.18	45.27 ± 0.58	72.74 ± 0.82	56.26 ± 0.74
C18:3, n-3	1.37 ± 0.15 ^b^	nd	11.20 ± 0.09 ^a^	0.31 ± 0.00 ^d^	0.51 ± 0.01 ^c^	nd
C18:3, n-6	nd	24.36 ± 0.96 ^a^	2.30 ± 0.05 ^b^	nd	nd	nd
C20:0	nd	nd	0.48 ± 0.00 ^a^	0.47 ± 0.01 ^a^	nd	nd
C20:1	nd	3.68 ± 0.28	nd	nd	nd	nd
C21:0	nd	nd	nd	nd	nd	0.72 ± 0.02 ^a^
C22:1	nd	3.08 ± 0.04 ^a^	2.35 ± 0.09 ^b^	nd	nd	nd

Explanatory notes: blue poppy seed (BPSO), borage (BSO), mustard seed (MSO), sesame (SO), safflower (SFO), and sea buckthorn (SBO) oils. The data in the table are presented as the mean ± standard deviation (SD) (*n* = 3). Differences between results for respective oils marked with the same letter in the same row are statistically insignificant (*p* < 0.05). The abbreviation “nd” indicates fatty acids that were not detected.

**Table 2:** The qualitative, nutritional, metabolic, and oxidizability indexes.

	MTSO	EPSO	FSO	CSSO	BCSO	PSO	BPSO	BSO	MSO	SO	SFO	SBO
	Qualitative indexes											
SFA	9.67 ^k^	12.05 ^g^	17.87 ^b^	12.31 ^f^	12.92 ^e^	21.18 ^a^	11.14 ^h^	14.28 ^d^	6.07 ^l^	15.49 ^c^	10.68 ^i^	9.84 ^j^
UFA	88.51 ^e^	87.94 ^f^	82.06 ^k^	82.67 ^j^	84.33 ^i^	77.85 ^l^	88.87 ^d^	85.73 ^g^	93.92 ^a^	84.51 ^h^	89.33 ^c^	90.17 ^b^
MUFA	26.42 ^e^	17.40 ^j^	20.90 ^i^	23.22 ^h^	27.62 ^d^	24.61 ^f^	15.33 ^l^	23.85 ^g^	51.34 ^a^	38.93 ^b^	16.08 ^k^	33.91 ^c^
PUFA	62.09 ^d^	70.54 ^c^	61.16 ^f^	59.45 ^g^	56.71 ^h^	53.24 ^j^	73.54 ^a^	61.88 ^e^	42.58 ^l^	45.58 ^k^	73.25 ^b^	56.26 ^i^
PUFA/SFA	6.42 ^d^	5.85 e	3.42 ^j^	4.83 ^g^	4.39 ^h^	2.51 ^l^	6.60 ^c^	4.33 ^i^	7.02 ^a^	2.94 ^k^	6.86 ^b^	5.72 ^f^
n-6/n-3	3.36 ^g^	292.92 ^a^	0.31 ^i^	0.13 ^j^	45.86 ^f^	63.14 ^d^	52.67 ^e^	-	2.80 ^h^	146.03 ^b^	142.62 ^c^	-
	Nutritional indexes											
AI	0.08 ^g^	0.10 ^e^	0.14 ^b^	0.10 ^d^	0.14 ^b^	0.18 ^a^	0.10 ^d^	0.12 ^c^	0.04 ^a^	0.13 ^c^	0.08 ^f^	0.07 ^g^
TI	0.08 ^d^	0.27 ^c^	0.11 ^d^	0.05 ^d^	0.26 ^c^	0.52 ^a^	0.23 ^c^	0.33 ^b^	0.07 ^d^	0.35 ^b^	0.23 ^c^	0.20 ^c^
h/H	12.98 ^c^	10.05 ^e^	7.07 ^k^	9.45 ^g^	7.32 ^j^	6.01 ^l^	9.60 ^f^	7.47 ^i^	26.28 ^a^	7.98 ^h^	12.09 ^d^	14.65 ^b^
	Metabolic indexes											
Elongase	-	37.87 ^g^	49.44 ^c^	4.26 ^j^	2.95 ^k^	65.87 ^a^	20.30 ^i^	35.10 ^h^	60.63 ^b^	42.10 ^f^	44.52 e	48.85 ^d^
Desaturase	85.38 ^g^	83.96 ^h^	78.34 ^k^	98.27 ^b^	98.77 ^a^	74.34 ^l^	89.08 ^f^	82.16 ^j^	95.86 ^c^	89.70 ^e^	83.01 ^i^	91.93 ^d^
ODR	70.53 ^f^	80.29 ^c^	74.64 ^d^	74.34 ^e^	68.22 ^i^	68.88 ^g^	82.75 ^a^	68.71 ^h^	45.18 ^l^	54.05 ^k^	81.99 ^b^	62.45 ^j^
LDR	22.98 ^d^	0.34 ^i^	76.59 ^b^	88.65 ^a^	2.13 ^e^	1.56 ^g^	1.86 ^f^	-	27.81 ^c^	0.68 ^h^	0.70 ^h^	-
	Oxidizability indexes											
UI	168.17 ^d^	158.72 ^g^	190.10 ^b^	203.52 ^a^	147.12 ^i^	132.03 ^k^	163.78 ^e^	171.97 ^c^	150.00 ^h^	130.40 ^l^	163.09 ^f^	146.43 ^j^
PI [%]	77.13 ^d^	71.21 ^g^	108.56 ^b^	112.66 ^a^	58.58 ^h^	54.67 ^k^	75.29 ^e^	86.67 ^c^	57.30 ^i^	46.86 ^l^	74.16 ^f^	57.11 ^j^
OI	0.77 ^c^	0.71 ^c^	1.08 ^a^	1.13 ^a^	0.58 ^d^	0.55 ^d^	0.75 ^c^	0.87 ^b^	0.57 ^d^	0.47 ^e^	0.74 ^c^	0.57 ^d^
Cox	8.28 ^d^	7.47 ^g^	11.80 ^b^	12.28 ^a^	6.24 ^i^	5.82 ^k^	7.88 ^e^	9.29 ^c^	6.40 ^h^	5.12 ^l^	7.76 ^f^	6.13 ^j^
OS	3612.11 ^d^	3204.90 ^g^	5351.50 ^b^	5598.47 ^a^	2646.59 ^i^	2466.17 ^k^	3399.98 ^e^	4148.25 ^c^	2709.94 ^h^	2107.08 ^l^	3340.38 ^f^	2565.61 ^j^

Explanatory notes: Atherogenicity index (AI), thrombogenicity index (TI), hypocholesterolemic/hypercholesterolemic ratio (h/H), linoleic (LDR) and oleic (ODR) desaturation ratios, unsaturation index (UI), peroxidizability index (PI), oxidizability index (OI), oxidizability value (Cox), oxidative susceptibility (OS). Differences between results for respective oils marked with the same letter in the same row are statistically insignificant (*p* < 0.05).

**Table 3:** The content of polyphenols in the investigated oils (mg/kg).

	MTSO	EPSO	FSO	CSSO	BCSO	PSO	BPSO	BSO	MSO	SO	SFO	SBO
	Flavonoids (mg/kg)											
Apigenin	84.74 ± 0.83 ^g^	111.7 ± 2.43 ^c^	75.08 ± 1.65 ^h^	86.3 ± 1.44 ^f^	89.93 ± 0.57 ^e^	55.16 ± 1.29 ^i^	54.2 ± 0.99 ^j^	125.17 ± 1.68 ^b^	37.76 ± 0.42 ^k^	25.46 ± 0.16 ^l^	126.4 ± 2.10 ^a^	102.57 ± 0.61 ^d^
Catechin	39.57 ± 0.79 ^f^	16.27 ± 0.16 ^i^	65.78 ± 1.14 ^b^	50.35 ± 0.45 ^d^	74.95 ± 0.53 ^a^	14.46 ± 0.11 ^k^	46.67 ± 0.66 ^e^	24.21 ± 0.30 ^g^	16.41 ± 0.31 ^h^	14.92 ± 0.23 ^j^	11.49 ± 0.22 ^l^	65.03 ± 0.98 ^c^
Kempferol	10.72 ± 0.09 ^l^	32.20 ± 0.47 ^f^	40.93 ± 0.39 ^c^	13.73 ± 0.30 ^j^	41.47 ± 0.29 ^b^	40.87 ± 0.9 ^d^	16.30 ± 0.13 ^i^	12.43 ± 0.23 ^k^	49.79 ± 1.23 ^a^	19.19 ± 0.16 ^h^	19.25 ± 0.32 ^g^	33.1 ± 0.63 ^e^
Luteolin	39.95 ± 0.77 ^f^	62.94 ± 0.80 ^b^	21.52 ± 0.25 ^k^	31.02 ± 0.47 ^h^	21.99 ± 0.21 ^j^	57.87 ± 0.72 ^d^	47.05 ± 0.45 ^e^	66.08 ± 1.20 ^a^	11.98 ± 0.22 ^l^	58.61 ± 1.12 ^c^	34.0 ± 0.59 ^g^	25.40 ± 0.3 ^i^
Naringenin	13.07 ± 0.30 ^g^	32.81 ± 0.17 ^e^	40.60 ± 0.87 ^c^	12.01 ± 0.22 ^i^	51.23 ± 0.70 ^a^	48.94 ± 0.81 ^b^	13.47 ± 0.16 ^f^	12.72 ± 0.19 ^h^	7.61 ± 0.08 ^l^	9.30 ± 0.08 ^k^	9.90 ± 0.22 ^j^	40.17 ± 0.22 ^d^
Quercetin	209.9 ± 4.99 ^a^	65.61 ± 1.52 ^j^	79.48 ± 0.70 ^g^	129.58 ± 0.95 ^c^	108.47 ± 2.44 ^e^	98.88 ± 2.44 ^f^	115.49 ± 1.49 ^d^	167.44 ± 2.24 ^b^	76.06 ± 0.94 ^i^	24.53 ± 0.59 ^l^	27.55 ± 0.64 ^k^	78.93 ± 1.94 ^h^
Rutin	18.52 ± 0.45 ^b^	12.82 ± 0.24 ^j^	14.24 ± 0.29 ^g^	15.66 ± 0.35 ^e^	14.44 ± 0.11 ^f^	16.09 ± 0.32 ^d^	13.44 ± 0.18 ^i^	12.83 ± 0.18 ^j^	18.69 ± 0.24 ^a^	12.17 ± 0.19 ^k^	16.55 ± 0.21 ^c^	14.12 ± 0.09 ^h^
Vitexin	34.10 ± 0.39 ^d^	29.21 ± 0.48 ^h^	30.97 ± 0.28 ^g^	33.60 ± 0.21 ^e^	51.13 ± 1.11 ^a^	40.74 ± 0.68 ^b^	32.98 ± 0.43 ^f^	34.4 ± 0.52 ^c^	3.49 ± 0.06 ^l^	12.02 ± 0.06 ^k^	16.83 ± 0.3 ^j^	26.71 ± 0.41 ^i^
Total	450.57 ± 10.03 ^c^	363.56 ± 4.60 ^h^	368.61 ± 2.89 ^g^	372.24 ± 8.27 ^f^	453.61 ± 6.42 ^b^	373.02 ± 7.90 ^e^	339.59 ± 2.91 ^i^	455.28 ± 2.79 ^a^	221.8 ± 4.97 ^k^	176.21 ± 1.44 ^l^	261.97 ± 4.61 ^j^	385.37 ± 7.65 ^d^
	Phenolic acids (mg/kg)											
4-hydroxybenzoic	168.71 ± 2.28 ^b^	nd	nd	196.17 ± 1.28 ^a^	96.24 ± 2.08 ^d^	55.91 ± 1.01 ^j^	135.99 ± 2.11 ^c^	91.18 ± 1.051 ^e^	63.53 ± 0.96 ^i^	30.08 ± 0.23 ^l^	55.46 ± 0.63 ^k^	83.56 ± 1.09 ^g^
Caffeic	86.92 ± 2.13 ^j^	194.81 ± 2.26 ^b^	166.01 ± 3.73 ^e^	77.78 ± 0.46 ^k^	189.38 ± 3.08 ^c^	76.41 ± 1.1 ^h^	84.6 ± 1.03 ^f^	117.19 ± 2.13 ^f^	92.59 ± 0.83 ^h^	112.2 ± 0.87 ^g^	36.17 ± 0.53 ^l^	170.37 ± 1.51 ^d^
Chlorogenic	76.49 ± 1.22 ^j^	332.43 ± 5.96 ^b^	402.62 ± 6.60 ^a^	65.75 ± 1.34 ^k^	279.22 ± 1.44 ^d^	233.61 ± 5.24 ^e^	81.94 ± 1.44 ^h^	58.28 ± 0.46 ^l^	114.82 ± 0.72 ^g^	76.74 ± 1.29 ^i^	121.59 ± 1.06 ^f^	297.44 ± 2.64 ^c^
Ferulic	65.58 ± 1.40 ^k^	119.00 ± 2.71 ^e^	119.44 ± 1.34 ^d^	292.8 ± 6.05 ^a^	136.23 ± 1.66 ^c^	145.56 ± 1.28 ^b^	74.86 ± 1.18 ^i^	86.25 ± 1.93 ^h^	13.75 ± 0.18 ^l^	115.74 ± 2.13 ^f^	74.13 ± 1.59 ^j^	111.67 ± 1.34 ^g^
Gallic	18.96 ± 0.49 ^h^	21.69 ± 0.18 ^b^	16.4 ± 0.37 ^k^	20.44 ± 0.31 ^c^	19.52 ± 0.34 ^d^	22.06 ± 0.34 ^a^	19.31 ± 0.35 ^f^	18.07 ± 0.13 ^j^	11.03 ± 0.16 ^l^	18.41 ± 0.41 ^i^	19.45 ± 0.28 ^e^	19.05 ± 0.27 ^g^
p-Coumaric	12.96 ± 0.17 ^i^	18.56 ± 0.17 ^e^	12.97 ± 0.18 ^i^	14.94 ± 0.11 ^g^	37.62 ± 0.65 ^a^	20.57 ± 0.21 ^b^	15.54 ± 0.31 ^f^	11.36 ± 0.25 ^k^	19.12 ± 0.31 ^d^	20.18 ± 0.16 ^c^	13.97 ± 0.24 ^h^	12.52 ± 0.11 ^j^
Protocatechuic	32.10 ± 0.25 ^j^	64.46 ± 0.43 ^d^	64.07 ± 0.80 ^e^	32.63 ± 0.81 ^i^	64.86 ± 0.93 ^b^	64.79 ± 0.71 ^c^	35.67 ± 0.23 ^h^	31.05 ± 0.52 ^l^	42.13 ± 0.61 ^f^	31.85 ± 0.57 ^k^	41.27 ± 0.65 ^g^	67.94 ± 1.12 ^a^
Sinapic	1629.96 ± 27.65 ^c^	854.27 ± 13.80 ^g^	788.53 ± 17.6 ^h^	1157.79 ± 28.04 ^d^	858.2 ± 12.52 ^f^	880.12 ± 5.09 ^e^	1838.48 ± 30.95 ^a^	1678.79 ± 27.21 ^b^	131.64 ± 2.39 ^l^	143.88 ± 1.81 ^j^	136.65 ± 3.27 ^k^	785.78 ± 7.46 ^i^
Syringic	94.71 ± 2.17 ^c^	74.44 ± 0.93 ^g^	69.18 ± 1.33 ^j^	73.63 ± 0.73 ^h^	73.5 ± 1.12 ^i^	76.34 ± 0.92 ^f^	86.52 ± 1.8 ^d^	49.08 ± 0.97 ^l^	117.68 ± 0.65 ^a^	84.29 ± 1.53 ^e^	114.23 ± 1.31 ^b^	68.2 ± 1.38 ^k^
t-Cinnamic	193.73 ± 1.37 ^b^	173.00 ± 2.84 ^f^	166.98 ± 3.48 ^g^	209.12 ± 1.45 ^a^	176.33 ± 1.26 ^d^	173.79 ± 2.71 ^e^	141.56 ± 1.61 ^h^	116.71 ± 1.93 ^i^	102.36 ± 0.56 ^l^	104.75 ± 1.13 ^k^	115.38 ± 0.81 ^j^	176.57 ± 1.45 ^c^
Vanillic	23.08 ± 0.42 ^g^	32.46 ± 0.41 ^c^	33.22 ± 0.19 ^a^	32.80 ± 0.51 ^b^	26.58 ± 0.26 ^f^	15.66 ± 0.35 ^j^	28.54 ± 0.67 ^e^	32.34 ± 0.61 ^d^	10.12 ± 0.21 ^l^	21.08 ± 0.33 ^i^	11.07 ± 0.25 ^k^	22.18 ± 0.44 ^h^
Vanillin	3.77 ± 0.08 ^b^	0.43 ± 0.01 ^i^	0.47 ± 0.01 ^i^	4.29 ± 0.04 ^a^	0.64 ± 0.01 ^h^	0.18 ± 0.01 ^j^	3.38 ± 0.02 ^c^	3.06 ± 0.05 ^d^	1.05 ± 0.02 ^g^	2.04 ± 0.03 ^e^	1.12 ± 0.01 ^f^	0.45 ± 0.01 ^i^
Total	2409.63 ± 19.50 ^b^	2061.96 ± 37.02 ^e^	1924.47 ± 19.98 ^g^	2181.8 ± 41.37 ^d^	1957.66 ± 47.97 ^f^	1886.45 ± 25.5 ^h^	2545.31 ± 43.76 ^a^	2305.69 ± 53.15 ^c^	719.82 ± 16.39 ^l^	761.24 ± 8.01 ^j^	740.51 ± 14.73 ^k^	1815.72 ± 32.11 ^i^
Total polyphenols	2860.21 ± 28.52 ^b^	2425.53 ± 16.89 ^e^	2293.08 ± 17.96 ^g^	2554.05 ± 59.63 ^d^	2411.27 ± 43.27 ^f^	2259.47 ± 30.36 ^h^	2884.89 ± 66.4 ^a^	2760.97 ± 36.69 ^c^	941.62 ± 7.64 ^k^	937.45 ± 21.77 ^l^	1002.47 ± 18.28 ^j^	2201.11 ± 52.22 ^i^

Results are expressed as mean ± SD (*n* = 3). Differences between results for respective oils marked with the same letter in the same row are statistically insignificant (*p* < 0.05). The abbreviation “nd” indicates “not detected”.

**Table 4:** The content of tocopherols and pigments in the investigated oils (mg/100 g).

	MTSO	EPSO	FSO	CSSO	BCSO	PSO	BPSO	BSO	MSO	SO	SFO	SBO
	Tocopherols (mg/100 g)											
α-T	20.47 ± 0.17 ^g^	10.63 ± 0.22 ^k^	4.23 ± 0.06 ^l^	44.87 ± 0.34 ^a^	18.33 ± 0.18 ^i^	35.03 ± 0.58 ^b^	29.32 ± 0.43 ^d^	16.79 ± 0.34 ^j^	19.23 ± 0.25 ^h^	26.07 ± 0.64 ^f^	27.48 ± 0.58 ^e^	34.6 ± 0.71 ^c^
β-T	0.30 ± 0.01 ^c^	0.17 ± 0.00 ^d^	nd	1.30 ± 0.02 ^b^	3.34 ± 0.05 ^a^	nd	nd	0.24 ± 0.01 ^c^	0.15 ± 0.00 ^d^	0.25 ± 0.02 ^c^	0.16 ± 0.01 ^d^	0.26 ± 0.01 ^c^
γ-T	35.47 ± 0.44 ^f^	32.77 ± 0.66 ^g^	79.93 ± 0.51 ^a^	10.37 ± 0.23 ^l^	25.63 ± 0.31 ^i^	11.61 ± 0.12 ^k^	27.09 ± 0.41 ^h^	36.61 ± 0.48 ^e^	17.85 ± 0.29 ^j^	38.36 ± 0.66 ^c^	36.74 ± 0.4 ^d^	75.02 ± 1.61 ^b^
δ-T	0.60 ± 0.01 ^e^	nd	nd	nd	1.30 ± 0.02 ^c^	13.63 ± 0.25 ^a^	0.56 ± 0.02 ^e^	0.34 ± 0.03 ^f^	0.65 ± 0.01 ^d^	0.17 ± 0.02 ^g^	1.55 ± 0.03 ^b^	nd
Total	56.24 ± 0.79 ^h^	43.57 ± 0.74 ^k^	84.16 ± 1.52 ^b^	56.54 ± 0.77 ^g^	48.6 ± 0.67 ^j^	60.27 ± 0.56 ^e^	56.97 ± 0.91 ^f^	53.98 ± 1.31 ^i^	37.88 ± 0.69 ^l^	64.85 ± 0.49 ^d^	65.93 ± 1.3 ^c^	109.88 ± 1.86 ^a^
	Carotenoids (mg/100 g)											
	0.31 ± 0.01 ^f^	0.44 ± 0.02 ^e^	1.35 ± 0.01 ^c^	0.47 ± 0.02 ^e^	0.70 ± 0.01 ^d^	8.96 ± 0.09 ^b^	0.13 ± 0.01 ^g^	0.45 ± 0.01 ^e^	0.71 ± 0.03 ^d^	0.10 ± 0.01 ^g^	0.47 ± 0.01 ^e^	229.22 ± 0.54 ^a^
	Chlorophylls (mg/100 g)											
	0.21 ± 0.01	0.17 ± 0.02 ^c^	0.06 ± 0.00 ^d^	0.18 ± 0.01 ^c^	0.89 ± 0.02 ^b^	21.14 ± 0.13 ^a^	0.04 ± 0.00 ^e^	0.83 ± 0.00 ^b^	0.06 ± 0.00 ^d^	0.02 ± 0.00 ^f^	0.02 ± 0.00 ^f^	0.86 ± 0.03 ^b^

Results are expressed as mean ± SD (*n* = 3). Differences between results for respective oils marked with the same letter in the same row are statistically insignificant (*p* < 0.05). The abbreviation “nd” indicates “not detected”.

**Table 5:** The growth rates of K_232_ and K_268_ after 10, 14, and 18 (or 21) * days of the test.

Days	MTSO	EPSO	FSO	CSSO	BCSO	PSO	BPSO	BSO	MSO	SO	SFO	SBO
	The growth rate of K_232_											
10	3.14 ^d^	3.92 ^c^	4.12 ^c^	5.92 ^b^	1.04 ^h^	1.22 ^g^	6.80 ^a^	3.76 ^c,d^	6.25 ^a^	1.47 ^f^	2.33 ^e^	2.24 ^e^
14	5.38 ^d^	7.50 ^c^	7.87 ^b^	7.87 ^b^	1.05 ^g^	1.21 ^g^	13.26 ^a^	8.03 ^b^	5.14 ^d^	1.96 ^f^	3.52 ^e^	3.46 ^e^
18 (or 21) *	5.84 ^g^	14.39 ^e^	33.17 ^a^	22.00 ^c^	1.43 ^j^	2.13 ^i^	25.26 ^b^	15.79 ^d^	12.98 ^f^	3.43 ^h^	8.64 ^g^	4.20 ^h^
	The growth rate of K_268_											
10	3.05 ^d^	18.58 ^b^	2.06 ^g^	24.40 ^a^	0.82 ^j^	0.82 ^j^	2.15 ^f^	3.67 ^c^	2.56 ^e^	1.35 ^h^	1.15 ^i^	1.10 ^i^
14	4.94 ^d^	39.12 ^b^	3.38 ^f^	47.05 ^a^	0.84 ^j^	0.80 ^j^	8.15 ^c^	8.13 ^c^	3.12 ^g^	4.38 ^e^	1.47 ^i^	1.58 ^h^
18 (or 21) *	11.68 ^f^	158.26 ^a^	19.10 ^d^	103.30 ^b^	0.77 ^k^	0.91 ^j^	20.74 ^c^	20.76 ^c^	13.67 ^e^	10.56 ^g^	4.57 ^h^	3.08 ^i^

Explanatory notes: (*) the growth rates of K_232_ and K_268_ for MSO, SBO, SFO, and EPSO were determined after 18 days; the growth rates of K_232_ and K_268_ for MTSO, FSO, CSSO, BCSO, PSO, BPSO, BSO, and SO were determined after 21 days, considered the final day of the test. Differences between results for respective oils marked with the same letter in the same row are statistically insignificant (*p* < 0.05).

**Table 6:** Correlation coefficients between the oils’ fatty acids profiles, chemical components (tocopherol, pigments, and TPC), and oxidative stability in thermostatic test and theoretical oxidizability index (Cox).

	K_232_	K_268_	10 Day (K_232_)	14 Day (K_232_)	18/21 Day (K_232_)	10 Day (K_268_)	14 Day (K_268_)	18/21 Day (K_268_)	Cox
C18:1	−0.37	−0.42	−0.07	−0.47	−0.40	−0.29	−0.33	−0.33	−0.52
C18:2	0.43	**0.59**	−0.31	−0.09	−0.44	−0.31	−0.25	−0.06	**−0.63**
C18:3	−0.15	−0.29	0.44	0.36	**0.67**	0.45	0.40	0.22	**0.91**
SFA	−0.33	−0.42	−0.45	−0.21	0.05	−0.10	−0.09	−0.09	0.09
MUFA	−0.42	−0.44	−0.02	−0.43	−0.37	−0.25	−0.29	−0.32	−0.45
PUFA	**0.66**	**0.71**	0.19	0.56	0.38	0.22	0.27	0.33	0.37
TPC	0.13	0.11	0.16	0.49	0.33	0.18	0.20	0.19	0.42
α-T	0.01	−0.12	−0.28	−0.13	0.09	**−0.72**	**−0.71**	**−0.63**	−0.15
γ-T	−0.11	0.09	0.08	0.04	−0.09	0.50	0.48	0.29	0.21
Total tocopherols	−0.20	−0.03	−0.18	−0.13	−0.13	0.09	0.07	−0.10	0.08
Carotenoids	−0.01	0.16	−0.22	−0.19	−0.27	−0.17	−0.18	−0.19	−0.25
Chlorophylls	−0.34	−0.23	−0.39	−0.39	−0.34	−0.19	−0.20	−0.20	−0.29
K_232_	1	**0.91**	0.51	**0.65**	0.49	0.23	0.29	0.49	0.14
K_268_		1	0.35	0.49	0.22	0.22	0.27	0.45	0.01
10-day (K_232_)			1	**0.85**	**0.76**	0.42	0.44	0.37	0.52
14-day (K_232_)				1	**0.83**	0.35	0.40	0.39	**0.59**
18/21-day (K_232_)					1	0.33	0.36	0.33	**0.79**
10-day (K_268_)						1	**0.99**	**0.92**	0.52
14-day (K_268_)							1	**0.94**	0.50
18/21-day (K_268_)								1	0.36
Cox									1

Red in bold—correlation statistically significant for *p* = 0.05.

**Table 7:** Principal component analysis (PCA) for the quality parameters of analyzed oils.

	PC1	PC2
C18:1, n-9*cis*	0.2153	0.2579
C18:2, n-6	0.0552	0.4239
C18:3, n-3	0.1898	0.3142
SFA	0.1080	0.0305
MUFA	0.2006	0.2881
PUFA	0.2372	0.3358
TPC	0.1599	0.1243
α-T	0.1652	0.1834
γ-T	0.2144	0.2549
Total	0.1019	0.1913
Carotenoids	0.1842	0.0631
Chlorophylls	0.1877	0.0729
K_232_	0.2208	0.3069
K_268_	0.2064	0.3339
10-day K_232_	0.2528	0.0652
14-day K_232_	0.2978	0.1016
18 (or 21)-day K_232_	0.2647	0.0134
10-day K_268_	0.2809	0.1778
14-day K_268_	0.2906	0.1456
18 (or 21)-day K_268_	0.2719	0.0312
Cox	0.2772	0.1550

## Data Availability

The original contributions presented in the study are included in the article; further inquiries can be directed to the corresponding authors.

## Supplementary Materials

The following supporting information can be downloaded at: https://www.mdpi.com/article/10.3390/molecules30153269/s1, Table S1: Determined values of specific absorbance coefficients, K_232_ and K_268_, during the storage test.
