# Supplementary material for: Nutritional Quality, Fatty Acids Profile, and Phytochemical Composition of Unconventional Vegetable Oils
Source: Molecules. 2025 Aug 4;30(15):3269. doi: 10.3390/molecules30153269 (PMC12348350; doi:10.3390/molecules30153269)
Supplement: Supplementary file 1 [file molecules-30-03269-s001.zip › molecules-3742905-supplementary.pdf]

Nutritional Quality, Fatty Acids Profile and Phytochemical Composition of Unconventional Vegetable Oils

Wiktoria Kamińska[1], Anna Grygier[2], Katarzyna Rzyska-Szczupak[3], Anna Przybylska-Balcerek[3], Kinga Stuper-Szablewska[3], Grażyna Neunert[1]*

[1] Department of Physics and Biophysics, Faculty of Food Science and Nutrition, Poznan University of Life Sciences, Wojska Polskiego 38/42, 60-637 Poznan, Poland; wiktoria.kaminska@up.poznan.pl (W.K.); grazyna.neunert@up.poznan.pl (G.N.)*;
[2] Department of Food Technology of Plant Origin, Faculty of Food Science and Nutrition, Poznan University of Life Sciences, Wojska Polskiego 31, 60-634 Poznan, Poland; anna.grygier@up.poznan.pl (A.G.);
[3] Department of Chemistry, Faculty of Forestry and Wood Technology, Poznań University of Life Sciences, 60-628 Poznań, Poland; anna.przybylska@up.poznan.pl (A.P-B); katarzyna.rzyska@up.poznan.pl (K.R-S); kinga.stuper@up.poznan.pl (K.S.-S.);
* Correspondence: grazyna.neunert@up.poznan.pl.

Table S1. Determined values of specific absorbance coefficients, K_{232} and K_{268}, during the storage test.

Storage Time [Day]	BPSO	BSO	MSO	SO	SFO	SBO
			K_{232}			
0	2.65±0.03^{e}	2.45±0.04^{f}	3.67±0.06^{d}	4.83±0.10^{c}	10.55±0.22^{b}	12.3±0.17^{a}
3	7.00±0.17^{d}	5.18±0.13^{e}	8.39±0.19^{c}	5.05±0.06^{f}	13.33±0.19^{a}	11.82±0.22^{b}
6	12.46±0.16^{c}	6.44±0.09^{e}	9.51±0.050^{d}	5.72±0.13^{f}	19.46±0.40^{b}	27.57±0.22^{a}
10	14.08±0.08^{d}	9.22±0.19^{e}	22.91±0.16^{b}	7.1±0.14^{f}	24.57±0.31^{a}	22.78±0.37^{c}
14	35.13±0.81^{c}	19.69±0.48^{d}	18.84±0.45^{e}	9.44±0.22^{f}	37.11±0.24^{b}	42.55±0.64^{a}
18	47.23±0.67^{c}	24.93±0.43^{e}	45.35±0.93^{d}	12.0±0.08^{f}	91.21±0.66^{a}	51.71±1.17^{b}
21	66.91±1.64^{a}	35.94±0.46^{c}	33.83±0.20^{d}	16.42±0.14^{f}	51.94±1.08^{b}	30.48±0.55^{e}
			K_{268}			
0	0.39±0.01^{d}	0.31±0.01^{e}	0.54±0.02^{c}	0.12±0.01^{f}	1.5±0.01^{b}	1.96±0.02^{a}
3	0.94±0.01^{d}	0.57±0.01^{e}	1.43±0.03^{a}	0.17±0.01^{f}	1.31±0.03^{b}	1.22±0.01^{c}
6	1.17±0.02^{c}	0.73±0.01^{e}	0.80±0.02^{d}	0.09±0.00^{f}	1.39±0.02^{b}	2.52±0.03^{a}
10	0.83±0.01^{e}	1.13±0.01^{d}	1.39±0.03^{c}	0.16±0.01^{f}	1.73±0.04^{b}	2.16±0.04^{a}
14	3.15±0.08^{a}	2.51±0.05^{c}	1.70±0.02^{e}	0.53±0.01^{f}	2.20±0.03^{d}	3.09±0.06^{b}
18	3.17±0.08^{e}	3.29±0.07^{d}	4.70±0.08^{c}	0.36±0.01^{f}	6.87±0.13^{a}	6.03±0.14^{b}
21	8.03±0.05^{b}	5.10±0.04^{d}	4.48±0.12^{e}	0.59±0.012^{f}	8.13±0.10^{a}	6.22±0.15^{c}

Explanatory notes: blue poppy seed (BPSO), borage (BSO), mustard seed (MSO), sesame (SO), safflower (SFO) and sea buckthorn (SBO) oils. The data in the table are presented as the mean ± standard deviation (SD). The data in the table are presented as the mean ± standard deviation (SD). Differences between results for respective oils marked with the same letter in the same row are statistically insignificant ($p < 0.05$).
